# Revitalizing cephalosporins: The promise of β-lactamase inhibitor combinations

**DOI:** 10.3205/dgkh000578

**Published:** 2025-09-09

**Authors:** Kazi-Chishti Marzooka, Shaikh Sajed, Mohamed Hassan Dehghan, Kazi Bilal, Rumani Imaan

**Affiliations:** 1 Department of Pharmaceutics, Maulana Azad Educational Trust’s, Y. B. Chavan College of Pharmacy, Dr. Rafiq Zakaria Campus, Aurangabad, Maharashtra, India; 2 Department of Hematology, Bone Marrow Transplantation and Cellular Therapy, SunAct Cancer Institute, Mumbai, India; 3 Department of Surgical Oncology, SunAct Cancer Institute, Mumbai, India

**Keywords:** beta-lactamase inhibitor combinations, gram-negative bacteria, antibiotic resistance

## Abstract

This review explore the rise of multidrug-resistant (MDR) Gram-negative bacteria, highlighting β-lactamase inhibitor combinations as crucial therapeutic options. It examines β-lactam resistance mechanisms, established combinations (e.g., ticarcillin/clavulanic acid, piperacillin/tazobactam), and the clinical efficacy of newer therapies like ceftazidime/avibactam (CAZ-AVI) for carbapenem-resistant *Klebsiella pneumoniae* (CRKP) and ceftolozane/tazobactam (TOL-TAZ) for MDR *Pseudomonas aeruginosa*. Additionally, novel combinations (e.g., cefepime-enmetazobactam, cefepime-taniborbactam) are discussed for tackling extensively drug-resistant (XDR) bacteria.

Through comparative analyses, this review provides key insights into efficacy, resistance, pharmacokinetics, and safety, guiding researchers in optimizing antimicrobial strategies and clinicians in managing MDR infections, while supporting antibiotic management and future research.

## Abstract (de)

Die Übersicht befasst sich mit der Zunahme multiresistenter (MDR) Gram-negativer Bakterien, wobei β-Lactamase-Inhibitor-Kombinationen als wichtige therapeutische Optionen hervorgehoben werden.

Untersucht werden β-Lactam-Resistenzmechanismen, etablierte Kombinationen (z.B. Ticarcillin/Clavulansäure, Piperacillin/Tazobactam) und die klinische Wirksamkeit neuerer Therapien wie Ceftazidim/Avibactam (CAZ-AVI) gegen Carbapenem-resistente *Klebsiella pneumoniae* (CRKP) und Ceftolozan/Tazobactam (TOL-TAZ) gegen MDR-*Pseudomonas aeruginosa*. Darüber hinaus werden neuartige Kombinationen (z.B. Cefepim-Enmetazobactam, Cefepim-Taniborbactam) zur Bekämpfung extensiv arzneimittelresistenter (XDR) Bakterien diskutiert.

Durch vergleichende Analysen liefert die Übersicht wichtige Erkenntnisse über Wirksamkeit, Resistenz, Pharmakokinetik und Sicherheit, die bei der Optimierung antimikrobieller Strategien und Klinikern bei der Behandlung von MDR-Infektionen helfen und gleichzeitig das Antibiotikamanagement und die zukünftige Forschung unterstützen.

## Introduction

β-Lactams are the most widely used antibiotics globally, and include various penicillin derivatives and related classes such as cephalosporins, cephamycins, carbapenems, monobactams, and penems [1], [2]. Despite their effectiveness, bacteria have developed resistance mechanisms, including the production of β-lactamase enzymes that break down the antibiotic structure. β-Lactamases are classified into four categories: A, B, C, and D, based on their structure and hydrolytic mechanisms. Class B enzymes, called metallo-β-lactamases (MBLs), require zinc ions for activity, while classes A, C, and D use serine [2], [3]. The overuse of β-lactams has contributed to the growing resistance to extended-spectrum cephalosporins and carbapenems, posing a serious global health threat. Particularly concerning are carbapenem-resistant bacteria, such as those encoding *Klebsiella* *(K.)* *pneumoniae* carbapenemase (KPC), New Delhi metallo-β-lactamase (NDM), and oxacillinase (OXA-48), due to their ability to resist multiple antibiotics. These growing resistances underscore the urgent need for effective antimicrobial strategies to address resistant bacterial infections and protect public health. A promising approach is the development of broad-spectrum β-lactamase inhibitors, designed to counteract β-lactam resistance by blocking the enzymes responsible for breaking down antibiotics like cephalosporins and carbapenems. These inhibitors target common pathogens treated with β-lactams, including *Escherichia (E.) coli*, *K. pneumoniae*, and *Pseudomonas (P.) aeruginosa* [1], [2], [3]. Resistance to β-lactams arises through various mechanisms, such as changes in membrane permeability, enzyme inactivation, and efflux pumps. Horizontal gene transfer, especially among carbapenemases-producing bacteria, plays a major role in spreading resistance [4], [5].This work aims to provide a detailed review of current β-lactamase inhibitors and their combinations, as well as explore newly developed or experimental compounds.

## Mechanism of action of β-lactam antibiotics and antibiotic resistance

β-Lactam antibiotics disrupt bacterial cell wall synthesis by mimicking the D-alanine-D-alanine structure in peptidoglycans, thereby inhibiting isopeptide bond formation by bacterial transpeptidases [6]. This interference reduces bacterial growth and division, while also weakening their defense against osmotic or tensile stress [7]. Penems, like faropenem, target L,D-transpeptidases, unlike carbapenems that focus on D,D-transpeptidases, making penems uniquely effective against mycobacteria [8], [9]. Penicillin-binding proteins (PBPs), essential for peptide cross-linking, are key targets of β-lactam antibiotics. PBPs are classified by molecular mass into various classes and subclasses [10], [11], [12], [13]. In Gram-negative bacteria, high-molecular-weight PBPs such as 1a, 1b, PBP2, and PBP3 play critical roles, and their inhibition can lead to cell death [14], [15].

**Figure 1:**
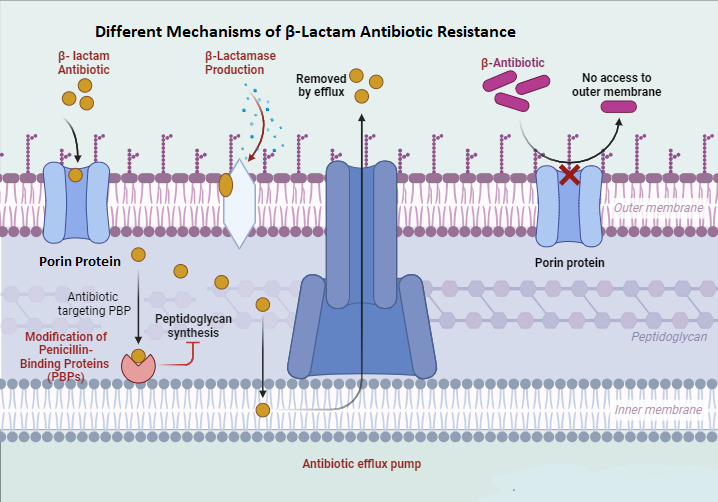
Resistance mechanism of the β-lactam antibiotic (created with BioRender.com)

For decades, β-lactam antibiotics have revolutionized the treatment of bacterial infections. However, their effectiveness is increasingly compromised by the rise and spread of resistance mechanisms, especially in Gram-negative bacteria. These mechanisms (Figure 1 (Fig. 1)) include the production of β-lactamase enzymes, such as extended-spectrum β-lactamases (ESBLs), which inactivate β-lactams and severely limit treatment options [16], [17]. Additionally, mutations in penicillin-binding proteins (PBPs) reduce β-lactams’ binding affinity, diminishing their effectiveness, making it crucial to identify specific PBP mutations for optimized treatment [18], [19]. Efflux pumps, membrane proteins that expel β-lactams from bacterial cells, also play a role in reducing drug efficacy and fostering resistance [20]. Furthermore, changes in the bacterial outer membrane, including altered porins and modifications to the lipopolysaccharide (LPS) layer, prevent β-lactams from entering the cell, lowering of periplasmic accumulation and therapeutic effectiveness [21], [22].

## β-Lactamase classification systems

The two primary classification systems for β-lactamases are the Ambler molecular classification and the Bush–Jacoby–Medeiros functional classification. These systems provide complementary information for understanding the enzymes that can inactivate β-lactam antibiotics [23], [24], [25].These classification are described in Table 1 (Tab. 1).

## β-Lactamase diversity in Gram-negative bacteria

The increasing diversity of β-lactamase genes in Gram-negative bacteria demandsthe continuous monitoring and development of new strategies to combat antibiotic resistance. Understanding these enzymes and their impact is key to preserving β-lactam effectiveness. Table 2 (Tab. 2) provides an overview of β-lactamase diversity in Gram-negative bacteria and strategies for combating antibiotic resistance for clinically relevant β-lactamase types and their activity spectrum.

## β-Lactam/β-lactamase inhibitor combinations in combating bacterial resistance

The following combinations are described: Ceftazidime-avibactam, ceftalozone-tazobactam, cefepime-zidebactam, cefepime-enmetazobactam and cefepime-taniborbactam.

### 1. Ceftazidime–avibactam(CAZ-AVI)

The misuse of antibiotics is fuelling a global health crisis, particularly concerning antimicrobial resistance (AMR), especially with CRKP [26], [27]. Carbapenems, once the last resort, are losing effectiveness against CRKP, leaving limited treatment options [26], [27]. This issue is especially alarming in countries such as China [28]. Urgent action is needed for new drugs to combat CRKP, as current strategies such as double-carbapenem therapy have limitations [29], [30]. Fortunately, a new generation of antibiotics, including plazomicin, eravacycline, meropenem-vaborbactam, and CAZ-AVI, is promising [31], [32]. The FDA approval of CAZ-AVI in February 2015 marked a milestone in the battle against challenging bacterial infections. This novel medication, combining a beta-lactamase inhibitor with a cephalosporin, offers a promising treatment option for complicated urinary tract and intra-abdominal infections. Its formulation addresses resistance mechanisms, offering new hope for effective treatment where traditional antibiotics have faltered. These medications, such as AVYCAZ^®^ (Allergan) and ZAVICEFTA^®^ (Pfizer) [33], have since become therapeutic options in the United States and are authorized for a range of serious infections caused by specific susceptible Gram-negative microorganisms in adults aged ≥18 years. Since its introduction on the Chinese market in September 2019, CAZ-AVI has garnered considerable attention for its proven clinical efficacy against carbapenem-resistant CRKPinfections. By inhibiting enzymes such asAmpC-producing beta-lactamase, ESBL, KPC, and OXA-48 carbapenemase, CAZ-AVI has emerged as a crucial weapon in China’s fight against CRKP, demonstrating its value as a vital addition to the global antimicrobial arsenal [34], [35].

#### Mode of action

Third-generation cephalosporins such as ceftazidime function similarly to other beta-lactam antibiotics. It works by attaching itself to PBPs and preventing bacterial cell wall peptidoglycan synthesis from occurring. Because of this disruption, proper cross-linking during the formation of the cell wall is prevented, which ultimately causes bacterial cell lysis and death [36], [37]. One of the first non-beta-lactam beta-lactamase inhibitors is avibactam. Despite having no inherent antibacterial activity, ceftazidime-avibactam plays a vital role in preventing ceftazidime from being broken down by different serine beta-lactamases [38], [39], [40]. Avibactam protects against ceftazidime via a gradual, reversible process of covalent acylation of beta-lactamase targets, which ultimately releases intact avibactam without hydrolysis. With the exception ofits activity against class B enzymes (MBL), its range of action includes Ambler class A (TEM-1, CTX-M-15, KPC-2, KPC-3), class C (AmpC), and certain class D beta-lactamases (OXA-48) [41], [42], [43].

#### Bacterial susceptibility and resistance profile

Different beta-lactamases, including class A ESBLs, class B carbapenemases, and class C cephalosporinases, can hydrolyse CAZ. Inhibiting class A, class C, and some class D beta-lactamases, such as CAZ and AVI, provides broad protection against Gram-negative bacteria. However, its effectiveness against Gram-positive bacteria, Gram-negative anaerobes, and isolates that produce class B beta-lactamases is limited [44], [45]. Clinical data show that CAZ-AVI is effective against isolates of enterobacterales that produce AmpC and ESBLs, among other beta-lactamase-producing bacteria. However, it has limited or no activity against OXA-24, OXA-40, and OXA-69 in *Acinetobacter (A.) baumannii*, and limited activity against OXA-48 in *K. pneumoniae*, which are specific class D carbapenemases. Global surveillance INFORM (International Network for Optimal Resistance Monitoring) studies revealed high susceptibility rates (99.5% to 100%) of enterobacteriaceae to CAZ-AVI, including isolates of *K. pneumoniae*, *Proteus (P.) mirabilis*, *E. coli*, and *K. oxytoca* that produce AmpC and ESBL [46].

Recent studies indicate that the combination of CAZ-AVI and aztreonam effectively combats resistant enterobacterisolates carrying the blaNDM-1 and blaKPC-4 genes. Avibactam’s resistance to various enzyme classes prevents NDM from hydrolyzing aztreonam, enhancing their synergistic effect. Additionally, this combination has successfully treated persistent bacteremia caused by *Stenotrophomonas maltophilia* with L1 (MBL) and L2 (cephalosporinase) beta-lactamases [47].While CAZ-AVI has been an effective option, the newly approved EMBLAVEO^®^ (aztreonam-avibactam) offers significant advantages. Unlike previous combinations, EMBLAVEO restores aztreonam’s activity against bacteria that co-produce MBLs and other β-lactamases more effectively, making it a superior choice for multidrug-resistant Gram-negative infections. The resistance profile of CAZ-AVI is outlined in Table 3 (Tab. 3). With this new formulation, clinicians can expect improved treatment outcomes and broader efficacy in tackling resistant infections.

#### Pharmacokinetic-pharmacodynamic (PK-PD) profile

CAZ and AVI exhibit comparable pharmacokinetic characteristics, such as short plasma half-lives, minimal binding to plasma proteins, and equivalent distribution within the epithelial lining fluid (ELF) [48], [49]. For both medications, the main route of elimination is renal excretion, as described in Table 4 (Tab. 4), while the key PK-PD profile is listed in Table 5 (Tab. 5).

The efficacy of CAZ-AVI in diverse patient populations and bacterial species is detailed in Table 6 (Tab. 6).

#### Safety profile and adverse events associated with CAZ-AVI usage

Compared with randomized controlled trials (RCTs), CAZ-AVI did not significantly differ in overall adverse event rates. However, CAZ-AVI was associated with a higher frequency of specific adverse events, including gastrointestinal issues (more than 20% of patients) and mild creatinine level elevations (≤2%). Additionally, 3% to 6% of patients experienced neurological adverse events, pyrexia, peripheral edema, hypersensitivity reactions, and other adverse events. Higher rates of serious adverse events (SAEs) were reported with CAZ-AVI, although detailed descriptions were lacking in the trials [50]. Nonrandomized studies have indicated that up to 5% of patients receiving CAZ-AVI experience acute kidney injury (AKI). Neurological and gastrointestinal side effects are also common, along with infrequent instances of leukopenia, rash, and abnormal liver function. However, these studies lacked thorough reporting of adverse events

### 2. Ceftalozone–tazobactam (TOL-TAZ)

The FDA and European Medicines Agency have approved TOL-TAZ, a novel combination of a β-lactamase inhibitor and a fifth-generation cephalosporin, for the treatment of several difficult adult infections. Officially approved for the treatment of complicated intra-abdominal infections, complicated urinary tract infections, pyelonephritis, and HABP, it has also demonstrated efficacy in the management of acute pulmonary cystic fibrosis exacerbations in adults; however, formal approval for this particular use has not been granted [51], [52].

#### Mode of action

Ceftolozane, a member of the cephalosporin class, shares structural similarities with ceftazidime. However, it differs in having a modified pyrazole side chain at the 3-position, which enhances its effectiveness against *P. aeruginosa*. Its mode of action involves targeting PBPs to inhibit bacterial cell-wall synthesis. Compared with ceftazidime, ceftolozane has a greater affinity for *P. aeruginosa* PBPs 1b, 1c, and 3 and shows greater stability against AmpC β-lactamase-mediated resistance, which is commonly observed in *P. aeruginosa* strains. Tazobactam, the β-lactamase inhibitor in this combination, irreversibly inhibits most class A and some class C β-lactamases. This extends the spectrum of ceftolozane activity to include most ESBL-producing Gram-negative bacteria and provides some activity against anaerobic organisms. The fixed-dose combination of ceftolozane and tazobactam is available at a 2:1 ratio, which has been determined to be the most potent combination through studies comparing various ratios (2:1, 4:1, and 8:1) [53].

#### Bacterial susceptibility and resistance profile

The antipseudomonal combination of TOL-TAZ is effective against a broad range of common Gram-negative pathogens. It has shown efficacy against various *Streptococcus* species, multidrug-resistant *P. aeruginosa*, ESBL-producing enterobacterales such as *E. coli* with CTX-M-14 and CTX-M-15, and some anaerobes such as *Bacteroides fragilis* and other non-Bacteroides Gram-negative bacteria [54], [55]. Ceftolozane is primarily responsible for the antibacterial effect against *P. aeruginosa*, with tazobactam contributing minimally. Tazobactam’s main function is to enhance ceftolozane’s activity against Enterobacteriaceae, although the combination shows relatively weak efficacy against *E. cloacae*. Bacterial susceptibility and resistance profiles are detailed in Table 7 (Tab. 7) and Table 8 (Tab. 8).

#### Pharmacokinetic-therapy and pharmacodynamic profile

The volume of distribution at the steady state (Vss) for TOL is 13.5 liters, whereas TAZ has a Vss of 18.2 liters. TOL has a longer half-life (3.12 hours) than TAZ (1.03 hours). TOL is 16–21% protein bound, and TAZ is 30% protein bound. TOL has an AUCELF/fAUC plasma ratio of 0.50, whereas TAZ has a ratio of 0.62. TOL has a renal clearance range of 57–112 ml/min, and TAZ has a renal clearance rate of 210 ml/min. TOL has a total clearance range of 68–112 ml/min, and TAZ has a total clearance of 340 ml/min. Compared with TAZ, TOL results in a lower Vss, longer half-life, lower percentage of protein binding, and distinct clearance patterns [56], [57], the key aspects of PK-PD are highlighted in Table 9 (Tab. 9). Thus, in combination therapy, the distinct pharmacokinetic profiles of TOL and TAZ offer complementary advantages, potentially enhancing the overall effectiveness of treatment.

The TOL-TAZ efficacy in diverse patient populations and bacterial species is detailed in Table 10 (Tab. 10).

#### Safety profile and adverse events

The safety profile of TOL-TAZ was generally comparable to that of the comparator in clinical trials. Common side effects included gastrointestinal issues, *C. difficile* infections, headaches, pyrexia, and abnormal liver function tests. Interestingly, the results of the trial in which high-dose TOL-TAZ was used for pneumonia suggested a greater risk of serious adverse events. In trials with a standard TOL-TAZ dose of 1.5 g every 8 hours, approximately 58% to 62% of the patients reported any adverse events, with 17.5% to 19% being drugrelated. Noteworthy side effects in these cases were gastrointestinal symptoms, sleeplessness, and abnormal liver function tests [58], [59]. In a study by Pogue et al. [60], 63% of 100 patients with MDR/XDR *P. aeruginosa* infections were on high-dose TOL-TAZ. Among the clinical outcomes, six cases of acute renal damage and four instances of *C. difficile* infection were noted, although safety data were not separately discussed. In a study conducted by Bassetti et al. [61], [62], drug-related adverse events were observed in 101 patients. Among the reported adverse events, gastrointestinal issues and abnormal liver function test results were identified as the primary concerns. Approximately 30% of these patients received high-dose TOL-TAZ.

### 3. Cefepime–zidebactam (CEP-ZID)

CEP and ZID (WCK 5222^®^) are unique combinations; zidebactam is a bicycloacyl hydrazide component that has built-in antibacterial action in addition to acting as a β-lactamase inhibitor. Zidebactam’s dual action protects cefepime against β-lactamase hydrolysis and simultaneously expands its antibacterial range. The in-vitro effectiveness of CEP–ZID and other antibacterial agents on a panel of clinical isolates that were well characterized and resistant to several drugs was recently examined. This investigation focused especially on a variety of carbapenemase manufacturers, resulting in a thorough evaluation of the combination’s efficacy against challenging Gram-negative isolates. The studyelucidated the efficacy of CEP-ZID in light of changing resistance patterns and new threats from MDR bacteria [63].

Although ZID is a non-β-lactam compound with inhibitory effects on class A and MBLs and targets PBP2, it still belongs to the diazabicyclooctane (DBO) class, much like avibactam. Unlike the four recently approved β-lactam/β-lactamase inhibitor combinations, ZID uniquely impacts MBLs. While CEP-ZID has shown promising in-vitro activity against MBL-positive pathogens, it is not yet approved for clinical use. Recent studies have shown a notable 90% to 100% susceptibility to CEP-ZID among 35 MBL-positive CPE strains, including those co-producing serine β-lactamases [64]. The rise of MDR Gram-negative bacteria is a major threat. CEP kills bacteria by disabling cell-wall formation, whereas ZID protects CEP from breakdown and potentially aids in cell-wall disruption. This synergy broadens the effectiveness of CEP-ZID against resistant bacteria, including those with MBL enzymes, which is a growing concern. Early studies revealed promising activity against MDR bacteria, particularly MBL-positive strains. While not yet approved for clinical use, CEP-ZID’s potential as a weapon against MDR Gram-negative bacteria is significant. The bacterial susceptibility and resistance profile (CEP–ZID) [64], [65] is presented in Table 11 (Tab. 11) and Table 12 (Tab. 12).

#### Pharmacokinetic and pharmacodynamic characteristics

The pharmacokinetic analysis provided by the manufacturer for WCK 5222, indicates that ZID has a half-life of 1.84 to 2.39 hours, while CEP has an average half-life of two hours. Both drugs are primarily excreted through the kidneys. ZID shows dose-proportional increases in AUC0-∞ and Cmax, whereas CEP exhibits linear pharmacokinetics across different doses. These findings offer valuable insights into the clinical dosing and safety of WCK 5222. However, no human PK-PD studies have been published for the CEP-ZID combination.

#### Efficacy and safety

Carbapenem-resistant *P. aeruginosa* infections, particularly those involving MBL-producing strains, are challenging to treat due to limited therapeutic options and high mortality rates. Traditional treatments like polymyxins are often hampered by poor pharmacokinetics and significant side effects. In one case involving an extensively drug-resistant (XDR) *P. aeruginosa* producing NDM, conventional therapies worsened the condition. However, the successful use of WCK 5222 as salvage therapy in a patient with acute T-cell leukemia demonstrated its efficacy, with the treatment showing consistent activity against XDR strains, including MBL producers, and resulting in a positive outcome with no adverse events [66]. Additionally, another case report highlights the compassionate use of WCK 5222 for treating a drug-resistant NDM-expressing* P. aeruginosa* infection in a patient with intra-abdominal infection-induced sepsis [67]. The use of CEP/ZID has also been reported for the successful treatment of sino-pulmonary infections and skull-based osteomyelitis caused by NDM-producing *P. aeruginosa* in a renal transplant recipient [68]; vide supra for its efficacy against resistant strains. Its efficacy in pediatric patients also holds promising hope in the treatment of PAN drug-resistant *P. aeruginosa* in lung empyema [69]. Furthermore, its in-vivo efficacy against carbapenem-resistant *A. baumannii* has been demonstrated in neutropenic murine pneumonia and thigh infection models [70], [71], [72], [73]. Studies have also reported its in-vivo activity against *K. pneumoniae* producing KPC and OXA-48-like enzymes in murine models [74].

### 4. Cefepime–enmetazobactam (CEP-ENZ)

In the ongoing global fight against antibiotic-resistant bacteria, particularly enterobacteriaceae, ENZ – formerly known as AAI101 – stands as a ground-breaking weapon. Driven by ESBLs, third-generation cephalosporin (3GC)-resistant Enterobacteriaceae are a major priority pathogen recognized by the World Health Organization. Each year, they cause an astounding 50 million severe infections globally [75], [76]. Class A β-lactamases that are resistant to tazobactam are successfully defeated by the new β-lactamase inhibitor ENZ, which works through a unique mechanism. The dynamic pairing of ENZ and the fourth-generation cephalosporin CEPdemonstrates potency comparable to meropenem and outperforms piperacillin-tazobactam against recent clinical isolates of enterobacteriaceae [76], [77]. As an empirical treatment to spare carbapenem usage, CEP-ENZ is effective against organisms coproducing OXA-48 β-lactamases and AmpC, especially in areas where ESBL-producing bacteria are common [78], [79].

The FDA, EMA and CMHP have approved Exblifep (Orchid Pharma), a CEP-ENZ combination to treat cUTIs in people ≥18 years of age, which is a noteworthy development in the battle against antibiotic resistance. The superiority of ENZ over piperacillin-tazobactam both in terms of clinical cure and microbiological eradication represents a major milestone, resulting in its historical approval. This achievement was observed during a worldwide phase-III study, highlighting the efficacy and potential impact of ENZ in combating antibiotic-resistant infections. It has an excellent success rate of 79.1% and a safety profile like that of piperacillin-tazobactam (58.9%) [80], [81], [82].

#### Mode of action

ENZ is a newly developed β-lactamase inhibitor with improved bacterial cell penetration and potency, attributed to the presence of a single methyl group, which differentiates it from tazobactam. Its neutral charge enhances its ability to penetrate the bacterial cell wall more effectively. The CEP-ENZ combination selectively inhibits many class A β-lactamases, including CTX-M, TEM, KPC and SHV. On the other hand, it does not obstruct carbapenemases or class D β-lactamases. When used alone, ENZ has no inhibitory effect on Gram-negative bacteria. The combination has shown in-vitro effectiveness against AmpC- and ESBL-producing Enterobacterales, as well as *P. aeruginosa*, with CEP being the key contributor to this activity [83]. ENZ dramatically increases cefepime’s therapeutic effectiveness, according to *in vivo* trials conducted on a mouse model of septicemia [84]. This combination offers a possible therapeutic option and supports “carbapenem sparing” approaches for infections caused by enterobacterales that produce ESBLs. Notably, ENZ has no antibacterial action against *S. Maltophilia* or *A. baumannii* [85].

#### Bacterial susceptibility and resistance profile

ENZ significantly enhances CEP’s potency against Enterobacteriaceae, particularly *K. pneumoniae* and *E. coli*, reducing MIC values by up to eightfold. While its efficacy against *P. aeruginosa* is limited, ENZ demonstrates superiority over tazobactam, especially for ESBL-producing *K. pneumoniae*. Clinical data reveal high susceptibility rates for CEP/ENZ against enterobacteriaceae, reaching 98.1%, with promising activity against ESBL-producing strains [86], [87], [88], [89]. However, its effectiveness against KPC-producing organisms remains limited. CEP/ENZ maintains high susceptibility rates (98.3% to 98.8%) even against 3GC-resistant strains, exhibiting robust activity against diverse resistance mechanisms such as ESBLs (susceptibility rates: 98.9% to 99.9%) [90]. An overview of regional disparities in Enterobacterales susceptibility to CEP/ENZ is presented in Table 13 (Tab. 13). Clinical trials for cUTIs reveal a complex efficacy profile, with susceptibility varying due to the presence of multiple beta-lactamase genes and resistance mechanisms. No cross-resistance with non-beta-lactam antibiotics has been noted, suggesting a potential option for carbapenem- and cephalosporin-resistant infections. However, susceptibility testing remains essential for treatment decisions [91].

#### Pharmacokinetic and pharmacodynamic characteristics

The PK profiles of CEP and ENZ followed a standard two-compartment model with time-delineated intravenous input and first-order clearance. However, a prolonged terminal gamma phase of drug elimination was noted for CEP, requiring the addition of a third compartment for accurate representation of drug concentrations at later time points. In murine models, both drugs readily penetrate the epithelial lining fluid (ELF) of the lungs, without evidence of system hysteresis. Combination therapy with CEP/ENZ showed promising efficacy, especially against *K. pneumoniae*, with dose-response relationships established through murine experiments. Fractionation studies revealed no significant differences in antibacterial activity across different ENZ schedules. These findings support the potential of CEP/ENZ as a therapeutic option against multidrug-resistant pathogens, highlighting the importance of considering pharmacokinetics and pharmacodynamics in treatment optimization [92]. The PKPD characteristics of CEP/ENZ are discussed in Table 14 (Tab. 14), and the key aspects of PK-PD are articulated in Table 15 (Tab. 15).

#### Safety profile and adverse events

The adverse effects of CEP-ENZ share many of the common side-effects seen with other β-lactam/β-lactamase inhibitor (BL/BLI) combinations such as Avycaz^®^, Emblaveo^®^, or Zerbaxa^®^. The combination has significantly advanced the treatment of cUTIs and pneumonia in adults, although its use requires careful safety assessment by healthcare providers. While generally well tolerated, the combination may lead to serious adverse reactions, particularly in patients with a history of severe hypersensitivity to CEP or other beta-lactam antibiotics. Neurological complications, such as encephalopathy or seizures, are possible, especially in individuals with renal dysfunction, necessitating dose adjustments. Vigilance for *C. difficile*-associated diarrhoea (CDAD) is crucial because of the risk associated with antibiotic use. Other safety considerations include the potential for positive Coombs’ tests, alterations in blood clotting time, and the emergence of drug-resistant bacteria. Although clinical trials have reported manageable side effects such as elevated liver enzymes and mild allergic reactions, caution is advised in elderly patients and those with renal impairment, who may require potential dose adjustments. In the case of overdose, supportive care is recommended, with hemodialysis as a possible option to remove excess drug. Healthcare professionals should thoroughly evaluate potential adverse effects and patient suitability before prescribing the CEP/ENZ combination [91].

### 5. Cefepime–taniborbactam

Taniborbactam, initially known as VNRX-5133, was developed by Venatorx Pharmaceuticals in 2014, with a patent filed that same year. It is a cyclic boronate BLI, designed to combat antibiotic-resistant bacteria. Cefepime-taniborbactam, an investigational intravenous antibiotic combination, pairs CEP, a fourth-generation cephalosporin, with taniborbactam, a bicyclic boronate BLI that has a broad inhibitory profile against both serine-based and MBLs. This combination is being developed to treat HABP, VABP and cUTIs, including pyelonephritis. The U.S. FDA has accepted its new drug application and set a PDUFA date of February 22, 2024, particularly for review in the treatment of cUTIs.

A key feature of taniborbactam is its activity against various MBLs, unlike other BLIs. This strength, however, comes with a caveat – it should be reserved for cases where piperacillin/tazobactam or CEP/ENZ is not appropriate, and only if ceftazidime-avibactam proves ineffective. Multidrug-resistant Gram-negative bacteria, especially carbapenem-resistant Enterobacterales and *P. aeruginosa*, present a significant healthcare challenge due to resistance mechanisms, such as beta-lactamase production. Research has demonstrated taniborbactam’s broad inhibitory efficacy, restoring CEP’s antibacterial activity and offering a promising therapeutic option for combating MDR Gram-negative bacterial infections [93], [94], [95], [96].

#### Mode of action

CEP acts by inhibiting PBPs, particularly PBP3. PBPs are crucial enzymes responsible for cross-linking peptidoglycan polymers, which are vital components of the bacterial cell wall. By specifically binding to PBP3, CEP disrupts this cross-linking process, hindering the formation of a structurally sound cell wall. This ultimately leads to cell-wall instability and bacterial cell death.

Tanibactam, a potent beta-lactamase inhibitor, addresses a key resistance mechanism employed by MDR bacteria. Beta-lactamases are enzymes produced by some bacteria that can hydrolyse (breakdown) beta-lactam antibiotics, rendering them ineffective. Tanibactam specifically inhibits these beta-lactamases, preventing the degradation of CEP. This ensures that the antibiotic retains its bactericidal activity against MDR bacteria that otherwise possess beta-lactamase-mediated resistance [96].

The synergy between CEP and taniborbactam lies in their coordinated action against MDR bacteria. While CEP directly targets the bacterial cell wall, MDR bacteria may employ beta-lactamases to counter its effects. By inhibiting these beta-lactamases, tanibactam interferes with the bacterial defense mechanism. This enables CEP to exert its full bactericidal effect, overcoming resistance mechanisms and efficiently eliminating MDR bacteria. Together, CEP and taniborbactam form a potent combination, with CEP disrupting cell-wall synthesis and taniborbactam, ensuring its efficacy by preventing breakdown. This coordinated approach offers a significant advantage in combating MDR Gram-negative infections, effectively addressing established resistance mechanisms [96].

#### Bacterial susceptibility and resistance profile

**Figure 2:**
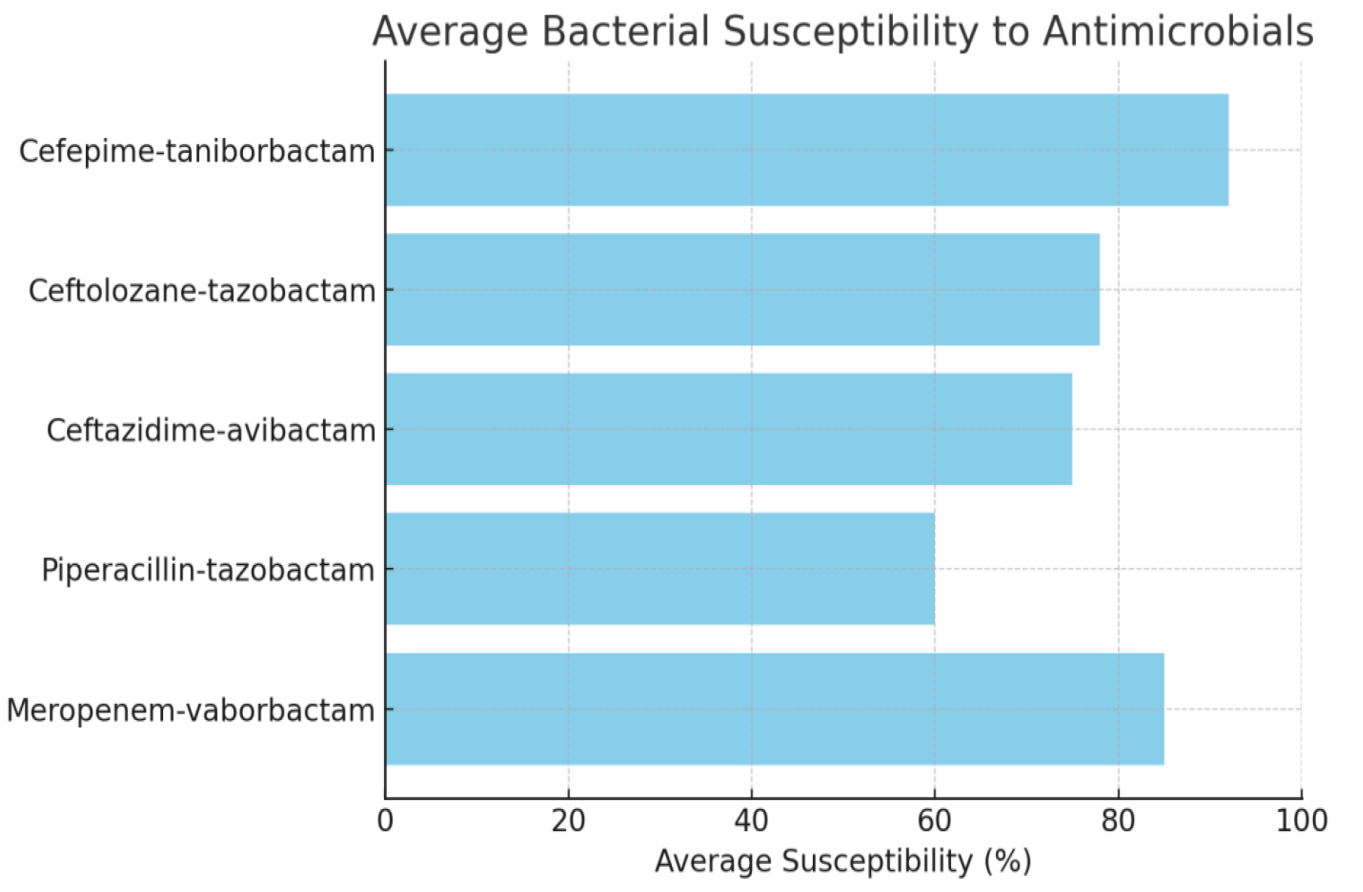
Average bacterial susceptibility toantimicrobials

Antimicrobial susceptibility studies have revealed alarming patterns of resistance in different bacterial phenotypes. CEP/taniborbactam is highly effective, showing near-universal susceptibility across studied multidrug-resistant bacterial strains. This is a positive advancement in the battle against antibiotic resistance (AMR). On the other hand, there has been an increase in resistance to well-known antibiotics, such as ceftolozane-tazobactam and piperacillin-tazobactam, especially in populations with ESBL or MDR phenotypes. This emphasizes how AMR is becoming a larger problem and how urgently new antibiotic drugs need to be researched and developed. The in-vitro susceptibility of multidrug-resistant Enterobacterales to CEP/taniborbactam compared with established antibiotics is depicted in Figure 2 (Fig. 2).

A recent study by Karlowsky et al. [97] evaluated the efficacy of CEP/taniborbactam against MDR Gram-negative bacteria. Conducted by IHMA and Venatorx Pharmaceuticals, the study analyzed strains of carbapenem-resistant Enterobacterales (CRE) and carbapenem-resistant Pseudomonas aeruginosa (CRPA) isolated from patients between 2018 and 2020. The results demonstrated a significant improvement in susceptibility rates compared to conventional β-lactam/β-lactamase inhibitor combinations, including ceftazidime-avibactam, ceftolozane-tazobactam, meropenem-vaborbactam, and piperacillin-tazobactam. Notably, CEP/taniborbactam exhibited greater than a 64-fold increased potency against Enterobacterales, with 99.7% of isolates inhibited at 16 mg/mL. Similar effectiveness was observed against *P. aeruginosa*, with a 4-fold increase in susceptibility and over 97% inhibition at the same concentration, even against strains with the VIM carbapenemase enzyme. The key bacterial susceptibility and resistance profiles are discussed in Table 16 (Tab. 16).

#### Pharmacokinetic and pharmacodynamic characteristics

Lasko et al. [98] demonstrated significant bacterial eradication in a neutropenic murine model of cUTI with CEP/taniborbactam, even against CEP-resistant clinical isolates harboring various enzymes, with an MIC of 32 mg/L. Human trials, including a phase-III study for cUTI treatment and safety assessment in healthy individuals, are ongoing [99]. Dowell et al. [100] found that taniborbactam when administered in human subjects exhibited dose-proportional pharmacokinetics, minimal accumulation, ~90% renal elimination, and good tolerability with no serious adverse events. A study focusing on lung penetration and efficacy against pneumonia analyzed the PK-PD profile of CEP/taniborbactam in twenty participants. Comparable plasma concentrations of taniborbactam and CEP before and after the third dose resulted in steady-state concentrations [101]. The findings of pharmacokinetics in plasma, ELF, and alveolar macrophages (AM) from a bronchoscopy study involving 20 participants are presented in Table 17 (Tab. 17).

Taniborbactam exhibited a 100% unbound fraction in plasma, with steady-state fAUC_0–8_ values consistent with previous phase-I findings. Cefepime, in contrast, showed a variable unbound fraction. Regarding lung penetration, taniborbactam demonstrated modest distribution into ELF and AM, with approximately 17% penetration efficiency into ELF and peak AM concentrations at 8 hours. For pharmacodynamics, the fAUC_0–24_:MIC ratio in free plasma is critical for taniborbactam’s efficacy against MDR pathogens. Abdelraouf et al. [102] identified the optimal PK/PD indices for cefepime-taniborbactam in murine thigh infection models, demonstrating that dosing frequency did not impact taniborbactam’s potentiation of cefepime, with fAUC_0–24_:MIC values supporting significant bacterial reduction against Enterobacterales and *P. aeruginosa*, and the HSR dose (0.5 g q8h) achieving ≥1 log kill against all test isolates.

#### Safety profile and adverse events associated

A recent clinical safety evaluation investigated the tolerability of CEP/taniborbactam, a promising antibiotic combination that targets MDR Gram-negative bacteria. The study included 20 subjects who received three doses of the drug. Notably, co-administration was well tolerated, with no reports of serious adverse events or fatalities. Treatment-emergent adverse events (TEAEs) were observed in 14 subjects, with the most frequent being a temporary increase in white blood cell count (leucocytosis), which affected six participants. Other TEAEs reported by at least two subjects included mild elevations in bilirubin levels, dizziness, chills, slight changes in kidney function, and variations in blood clotting parameters. Importantly, all TEAEs were classified as mild in severity, and no subject discontinued the study because of adverse effects [100].

Additionally, the bronchoscopy and bronchoalveolar lavage procedures used for lung assessment were well tolerated by all participants, with only two requiring minimal conscious sedation. These findings contribute to the growing body of evidence supporting a favorable safety profile for CEP/taniborbactam, suggesting its potential as a safe and effective therapeutic option for patients battling serious infections caused by MDR Gram-negative bacteria [99].

## Conclusion

The emergence of MDR Gram-negative bacteria, particularly with the rise of ESBLs, carbapenemases, and other β-lactamases, presents a significant challenge to antibiotic therapy. However, the development of novel β-lactam/β-lactamase inhibitor (BLBLI) combinations such as cefepime-zidebactam, cefepime-enmetazobactam, and cefepime-taniborbactam offers promising solutions. These combinations demonstrate potent antibacterial activity against various resistance mechanisms, providing effective treatment options for challenging pathogens such as Enterobacterales, *P. aeruginosa*, and *A. baumannii*. They also offer a carbapenem-sparing alternative for common infections caused by ESBL/AmpC-producing Enterobacterales and non-carbapenem-resistant *P. aeruginosa*. Despite their high cost, current recommendations suggest their use as definitive therapy for resistant isolates, with specific combinations preferred for certain resistant enterobacterales. Nevertheless, ongoing research and development of β-lactamase inhibitors are crucial to address the evolving landscape of antibiotic resistance. The emergence of new classes of β-lactamase inhibitors holds promise for protecting valuable antibiotics and overcoming resistance mechanisms. Overall, the development and implementation of novel BLBLI combinations represent significant progress in combating MDR Gram-negative bacteria, but continued efforts are essential to overcome emerging resistance threats.

## Notes

### Authors’ ORCIDs 

Marzooka KC: 0000-0002-6103-7806

Dehghan MH: 0000-0002-8082-9454

Bilal K: 0009-0000-8071-6268

Imman R: 0000-0001-9969-1128

### Funding

None. 

### Competing interests

The authors declare that they have no competing interests.

## Figures and Tables

**Table 1:**
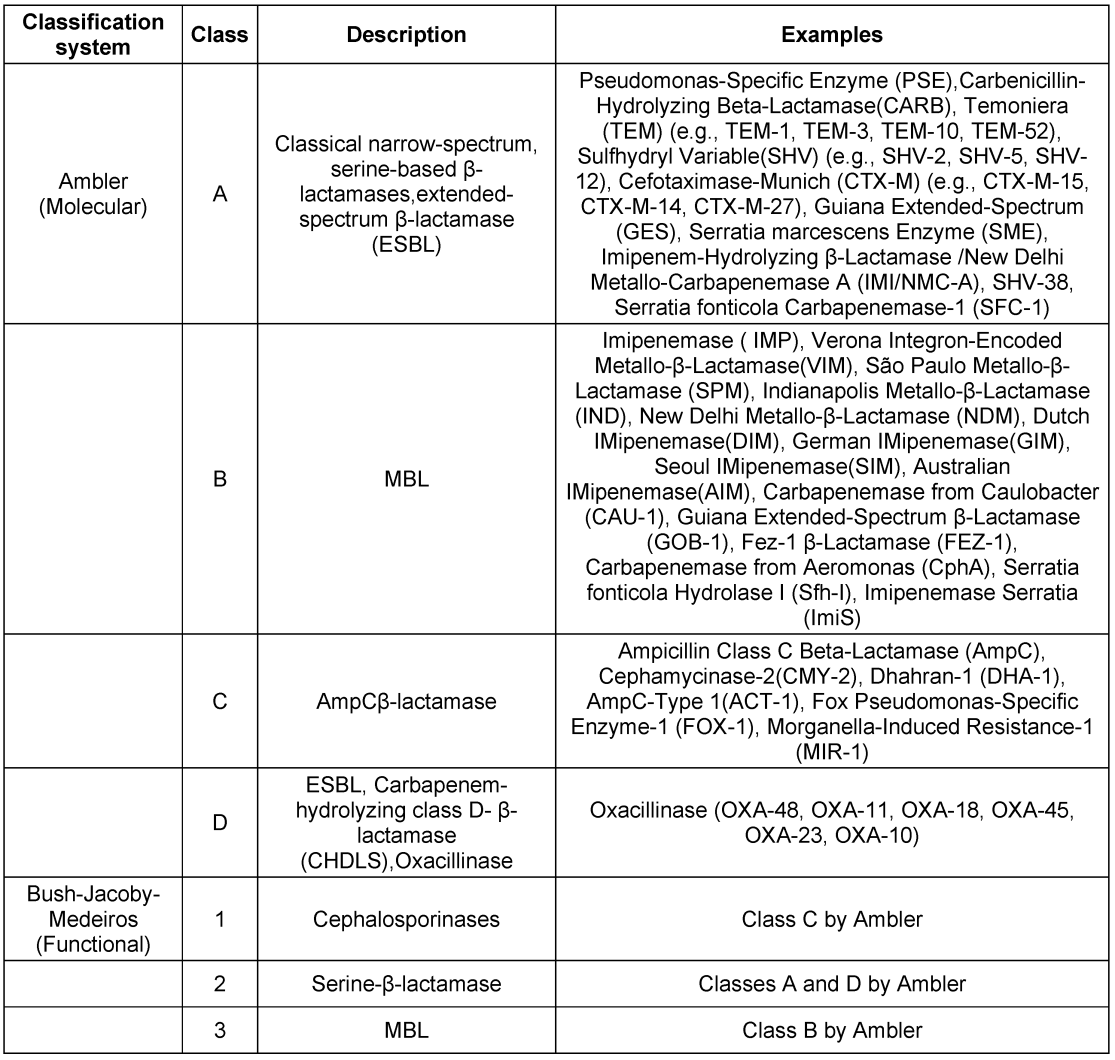
Classification of β-lactamases

**Table 2:**
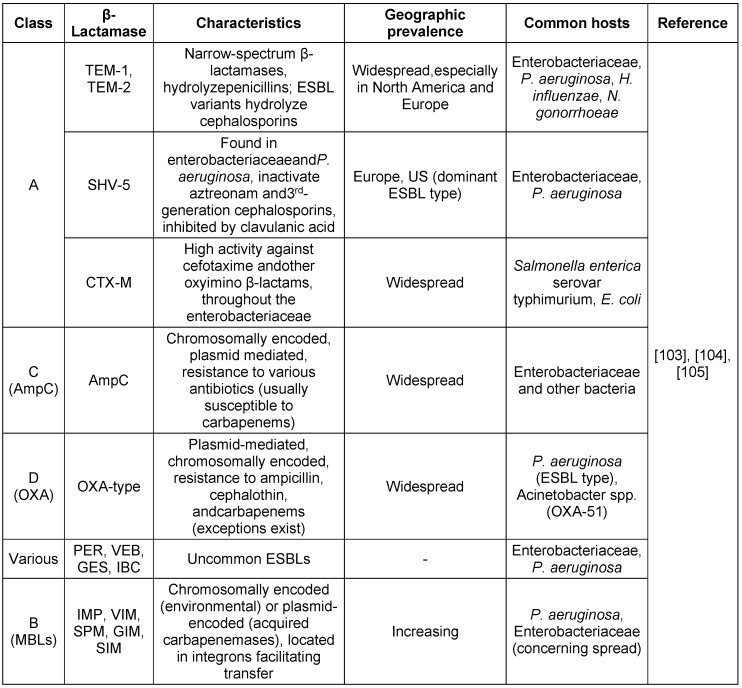
Clinically relevant β-lactamase types and their activity spectrum

**Table 3:**
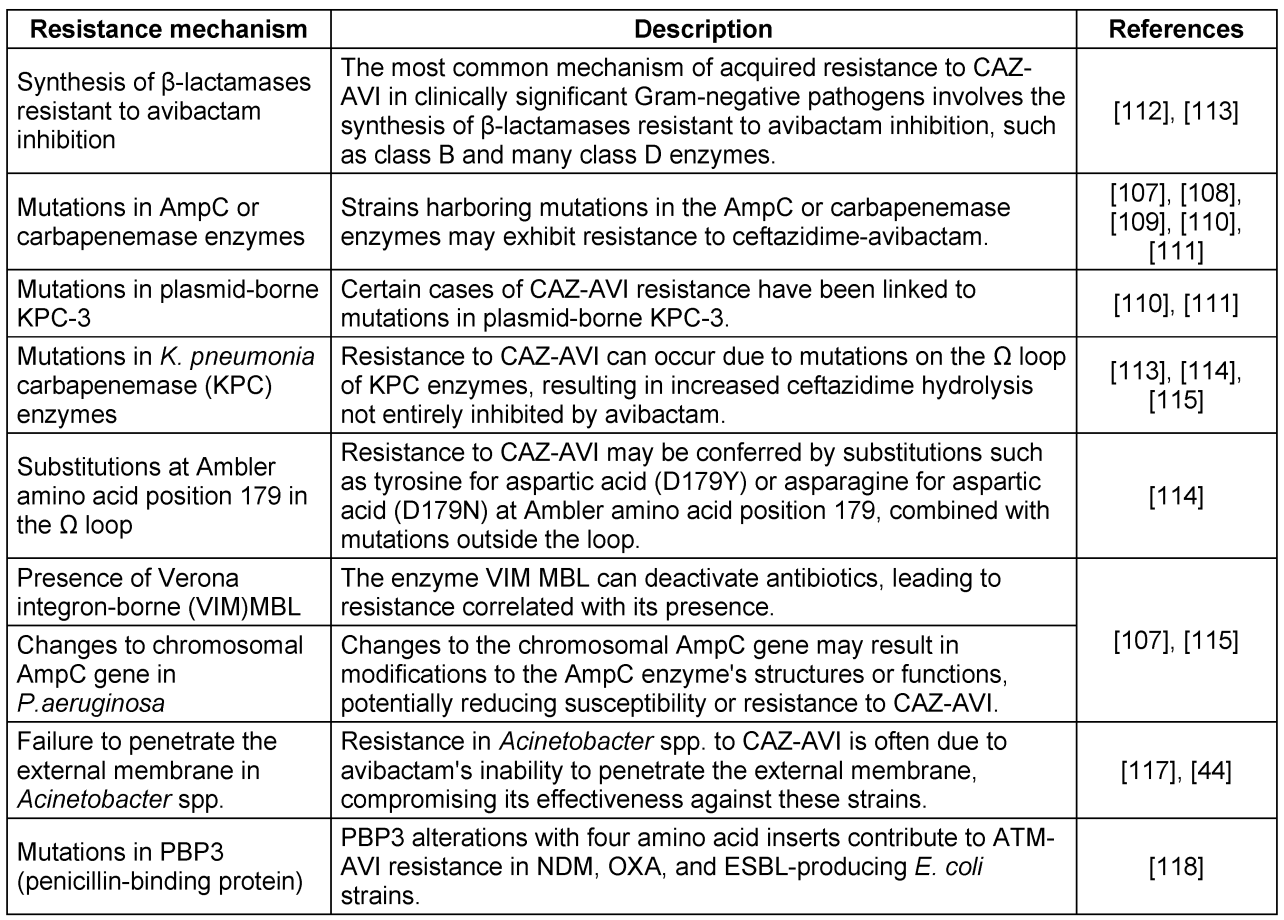
Resistance profile of ceftazidime-avibactam

**Table 4:**
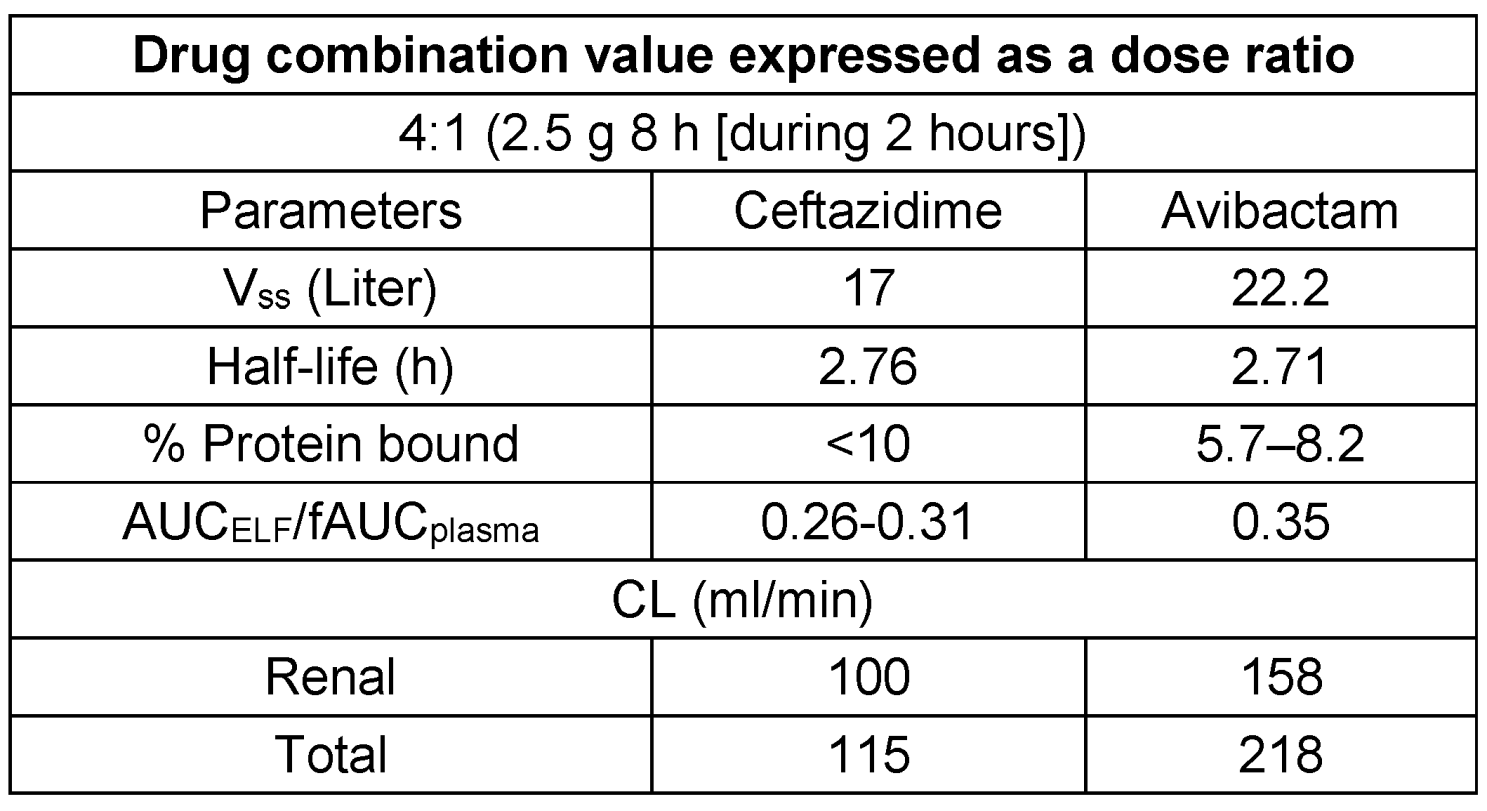
Pharmacokinetic characteristics of ceftazidime-avibactam [48], [49]

**Table 5:**
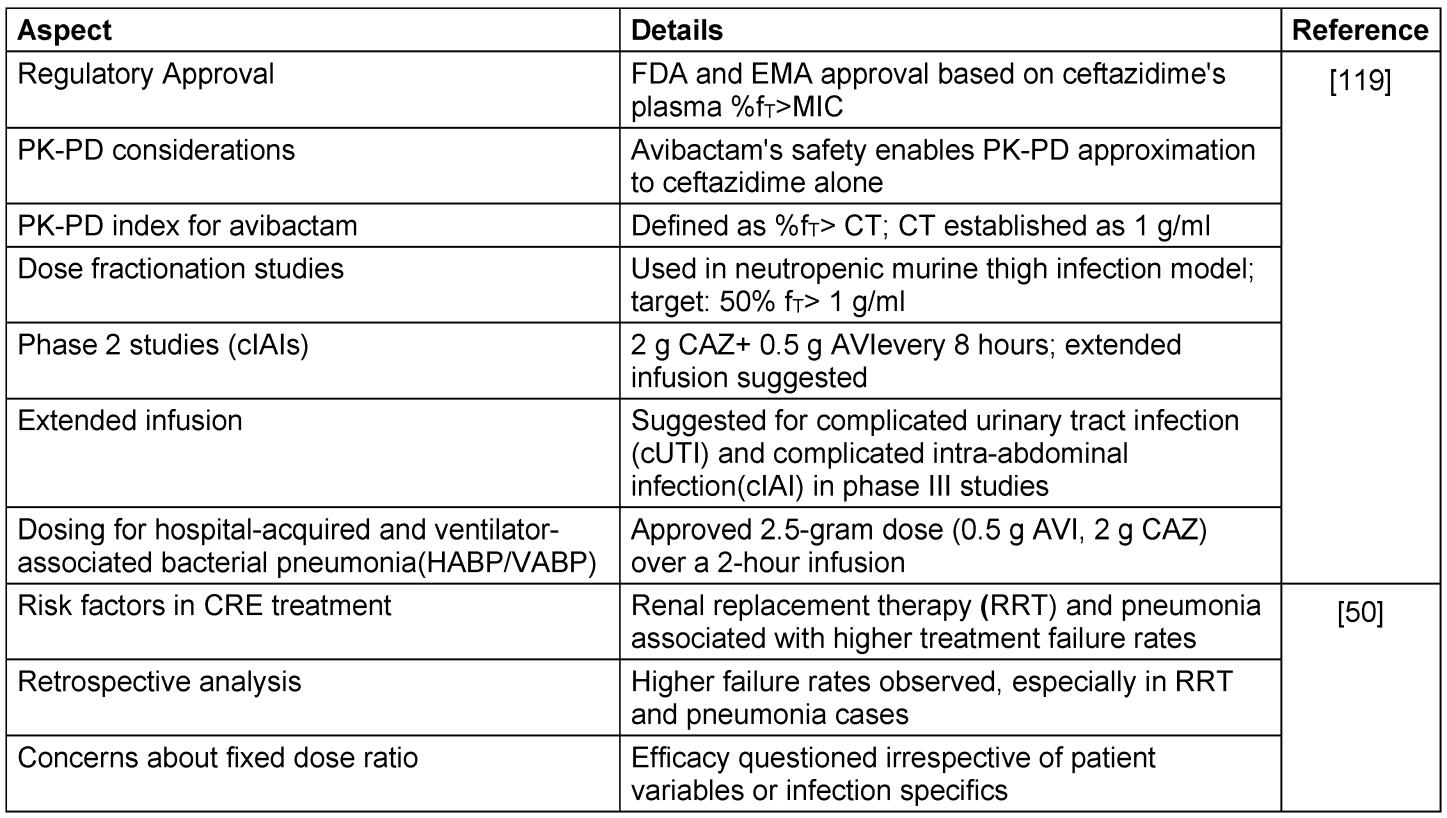
Ceftazidime-avibactam: Key aspects of PK-PD

**Table 6:**
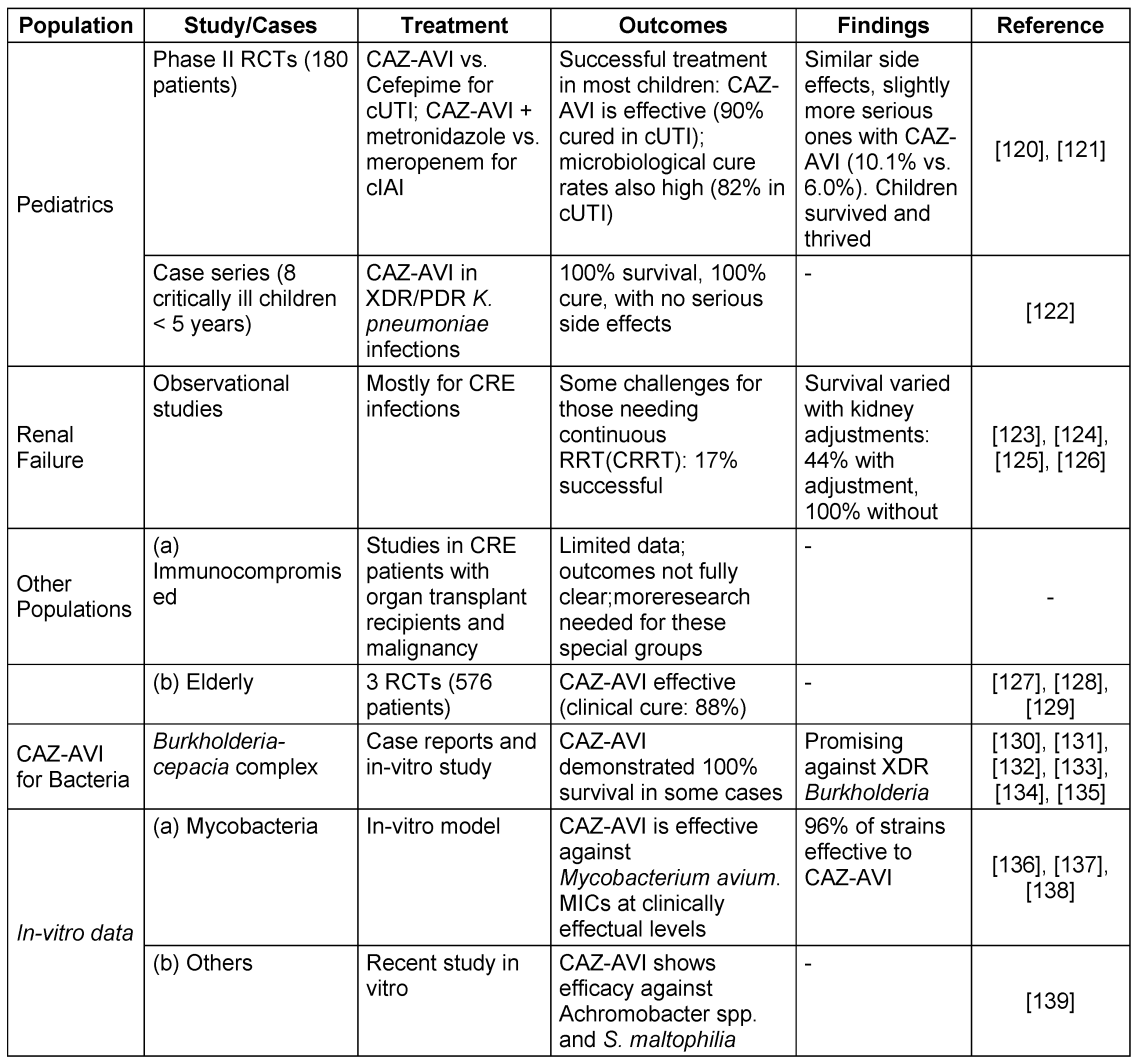
Ceftazidime-avibactam treatment outcomes in diverse patient populations

**Table 7:**
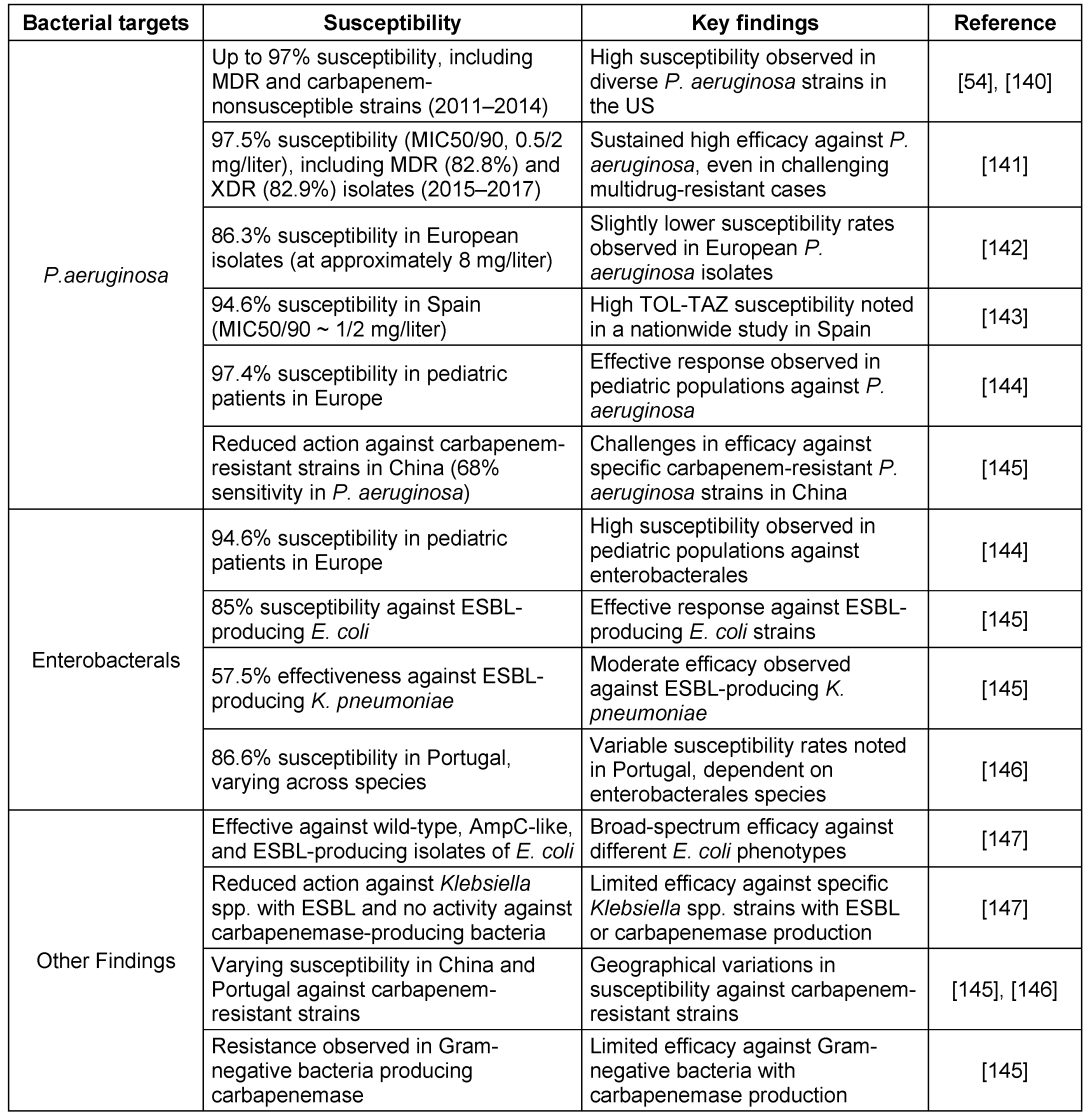
Ceftolozane-Tazobactam susceptibility in key bacterial populations

**Table 8:**
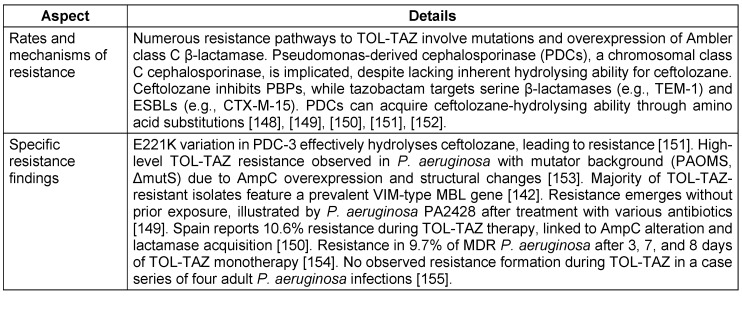
Resistance profile of Ceftolozane-Tazobactam

**Table 9:**
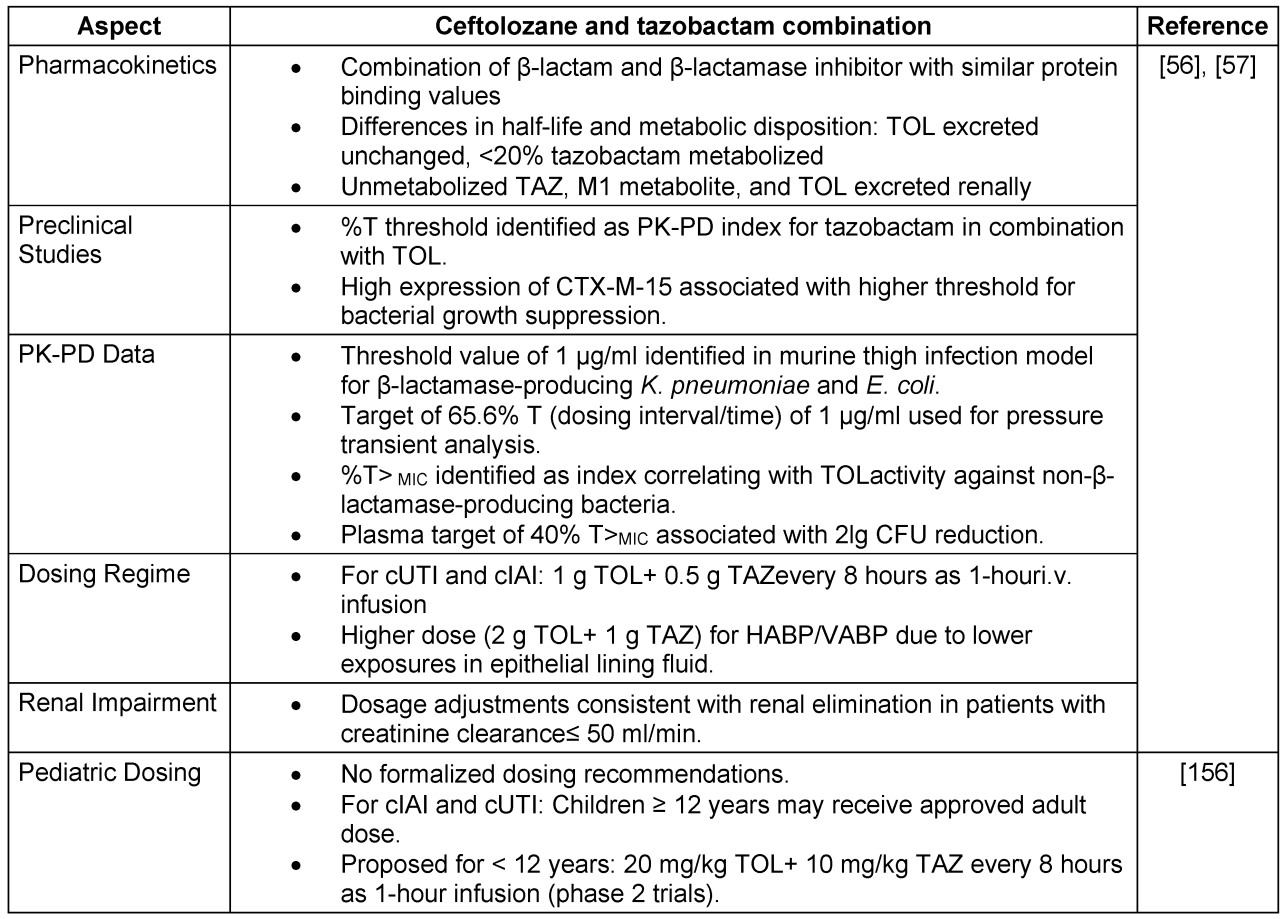
Key aspects of pharmacokinetic therapy and pharmacodynamic profile of Ceftolozane and tazobactam

**Table 10:**
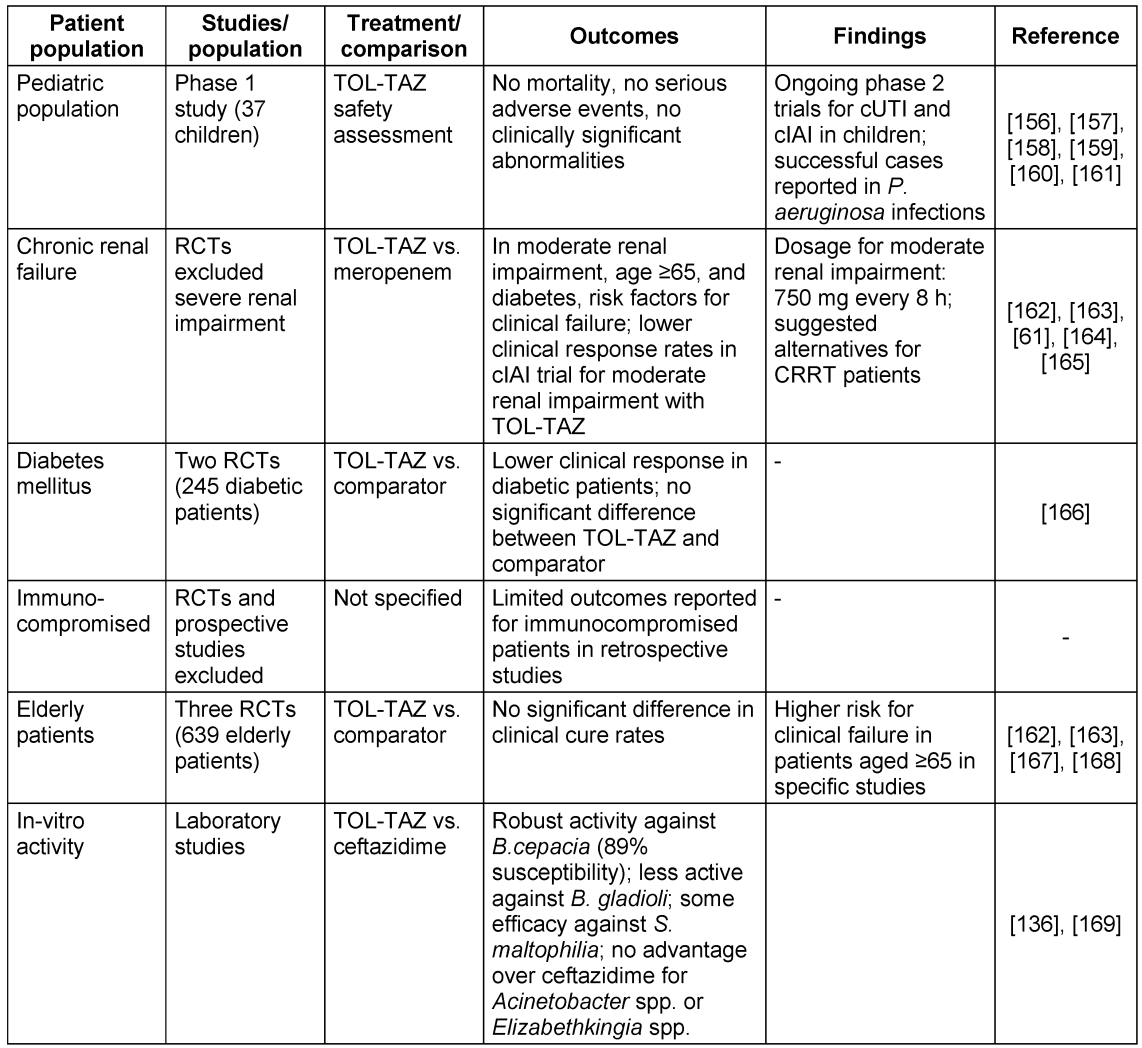
Ceftolozane and tazobactam treatment outcomes in diverse patient populations

**Table 11:**
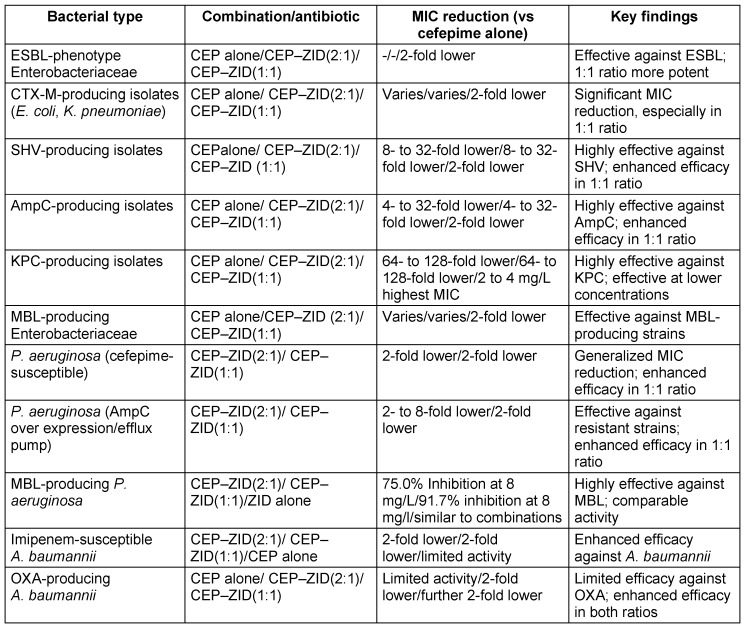
Cefepime/Zidebactam susceptibility in key bacterial populations (modified according to [65])

**Table 12:**
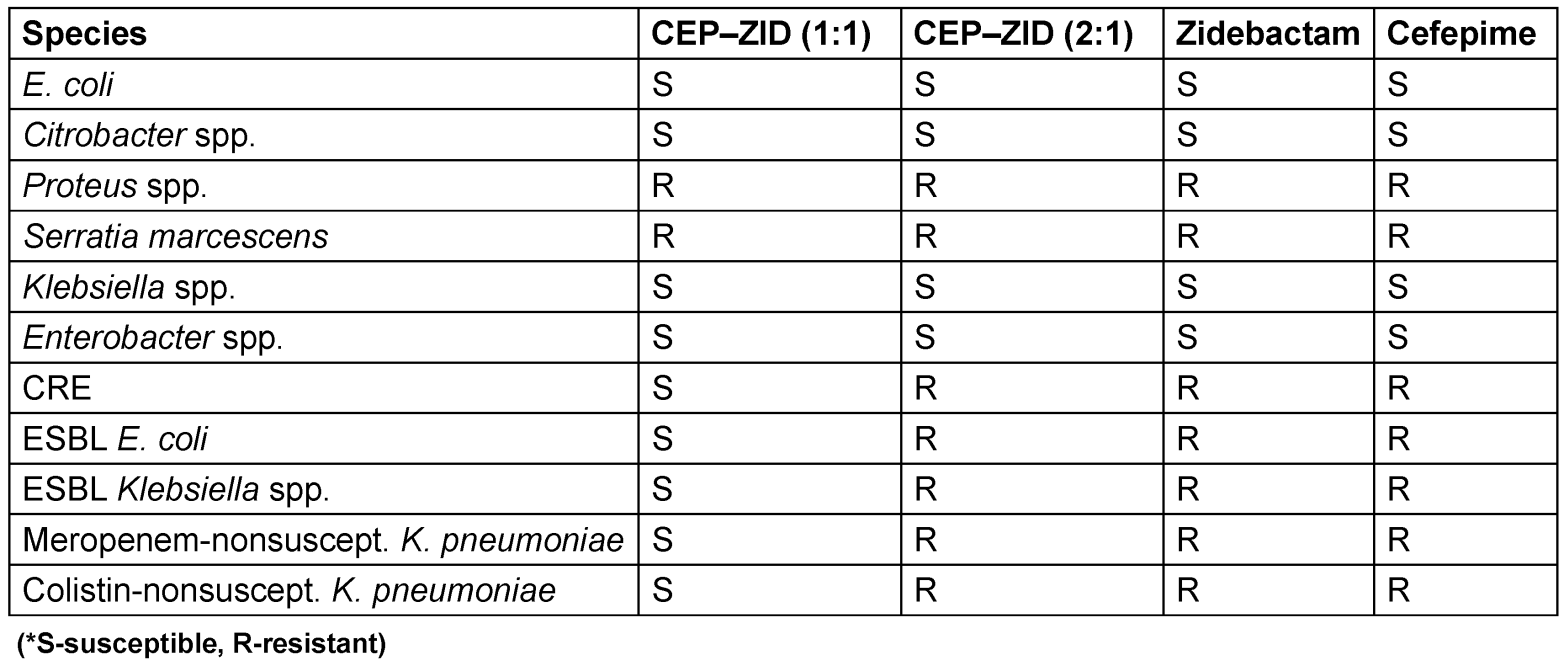
Resistance profile of cefepime-zidebactam

**Table 13:**
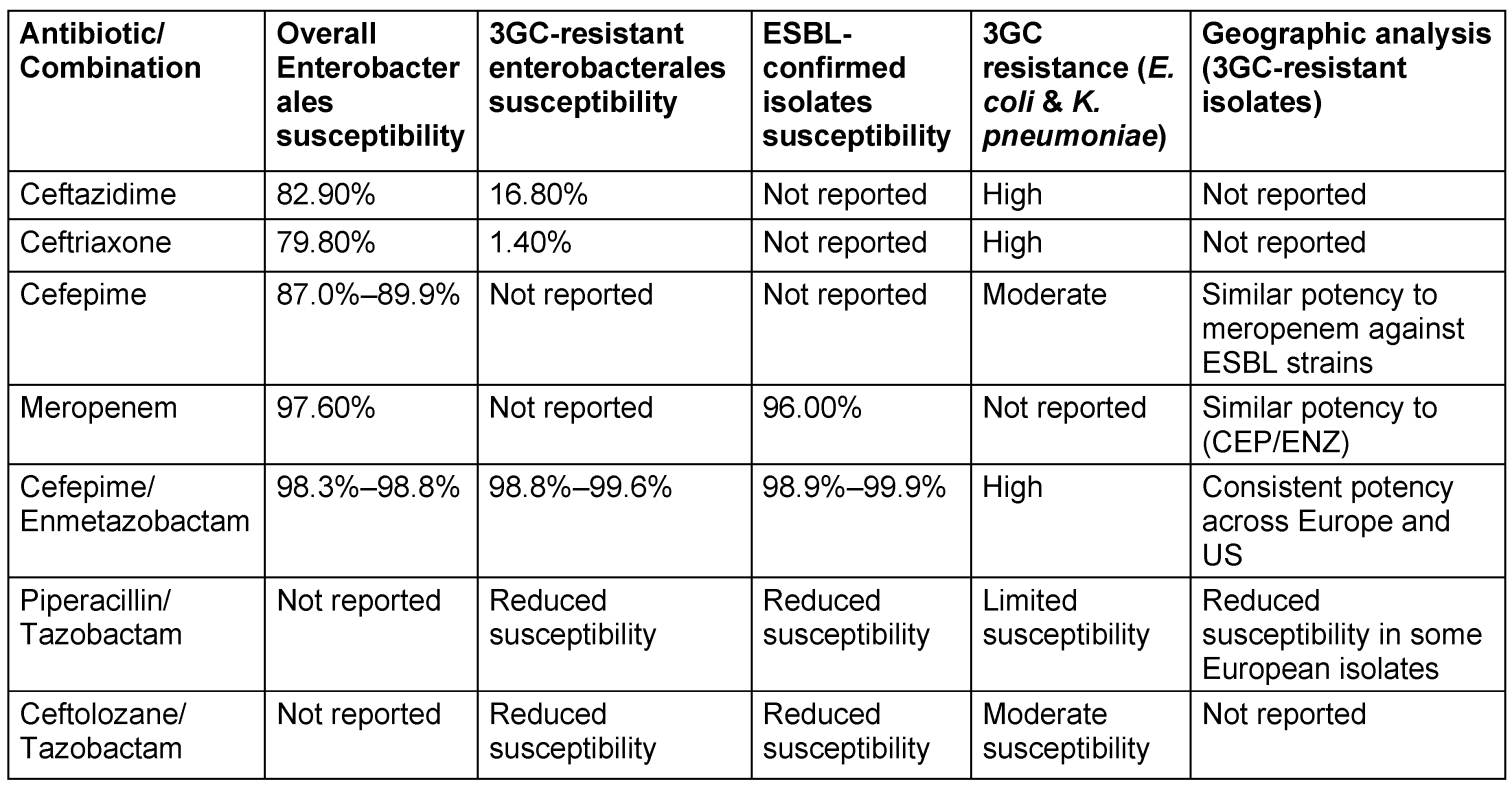
Geographic variations in the susceptibility of Enterobacterales to cefepime enmetazobactam (modified according to [90])

**Table 14:**
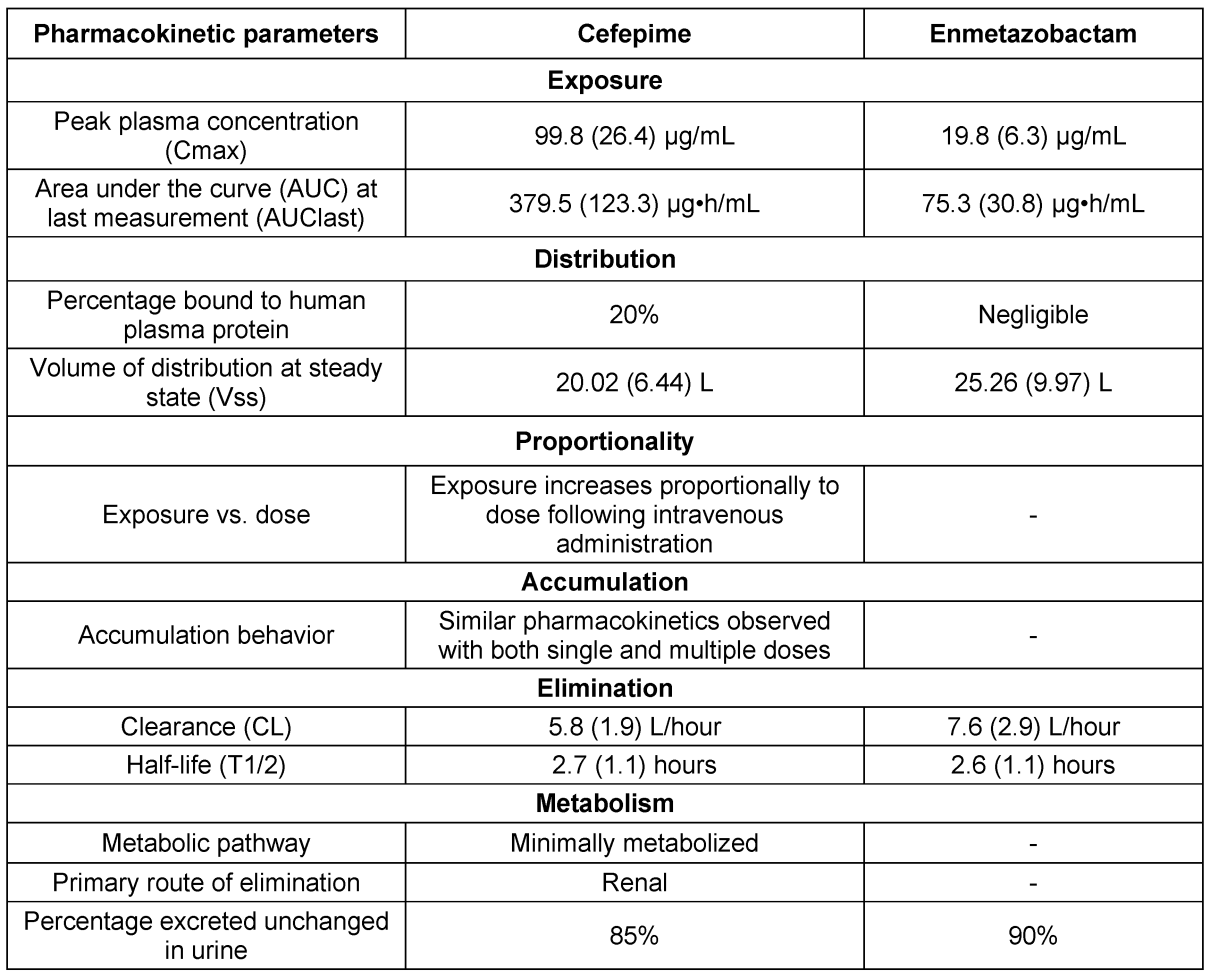
Pharmacokinetic parameters based on murine model data (modified according to [92])

**Table 15:**
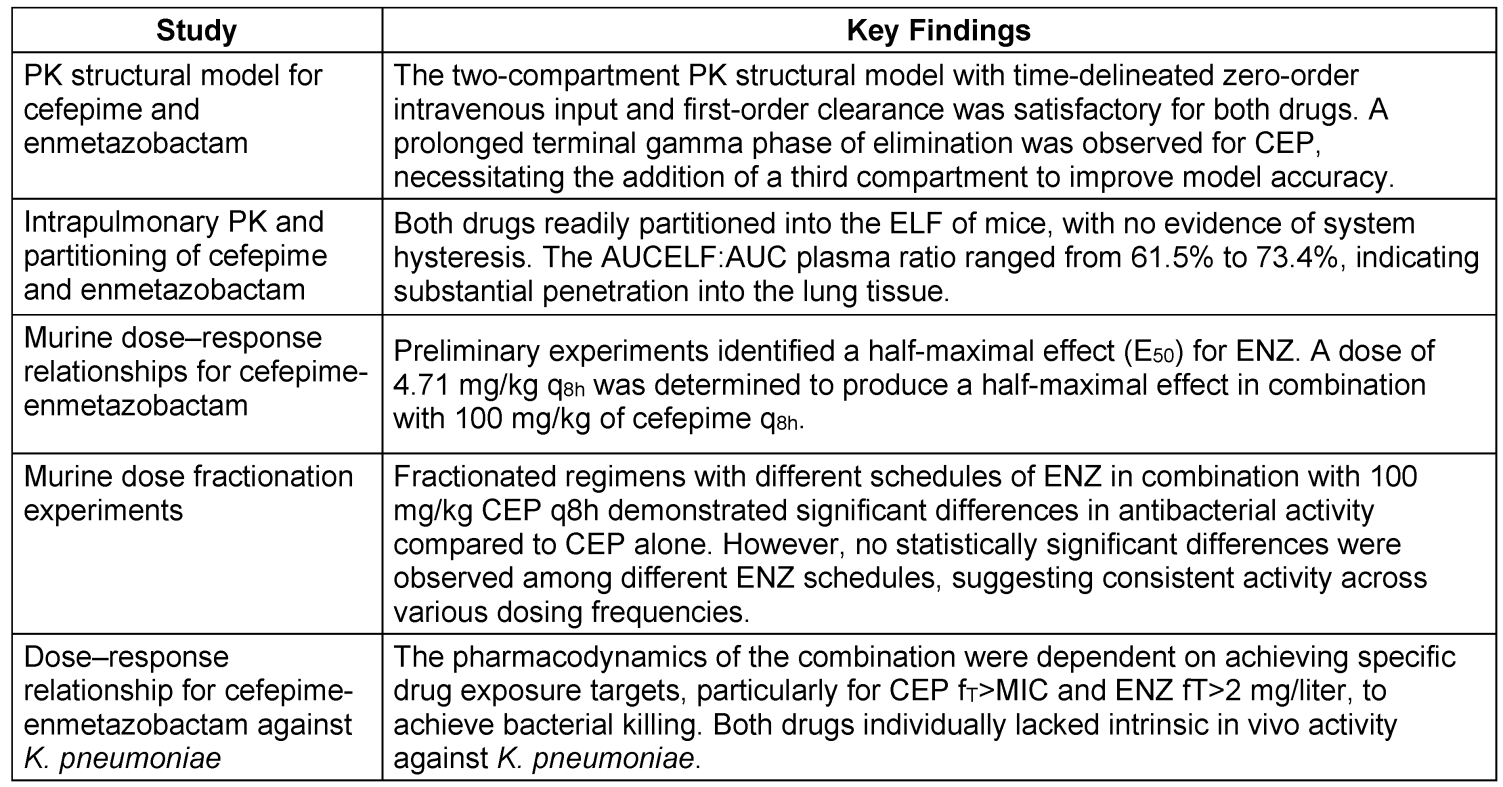
Key aspects of PK-PD of cefepime-enmetazobactam (modified according to [92])

**Table 16:**
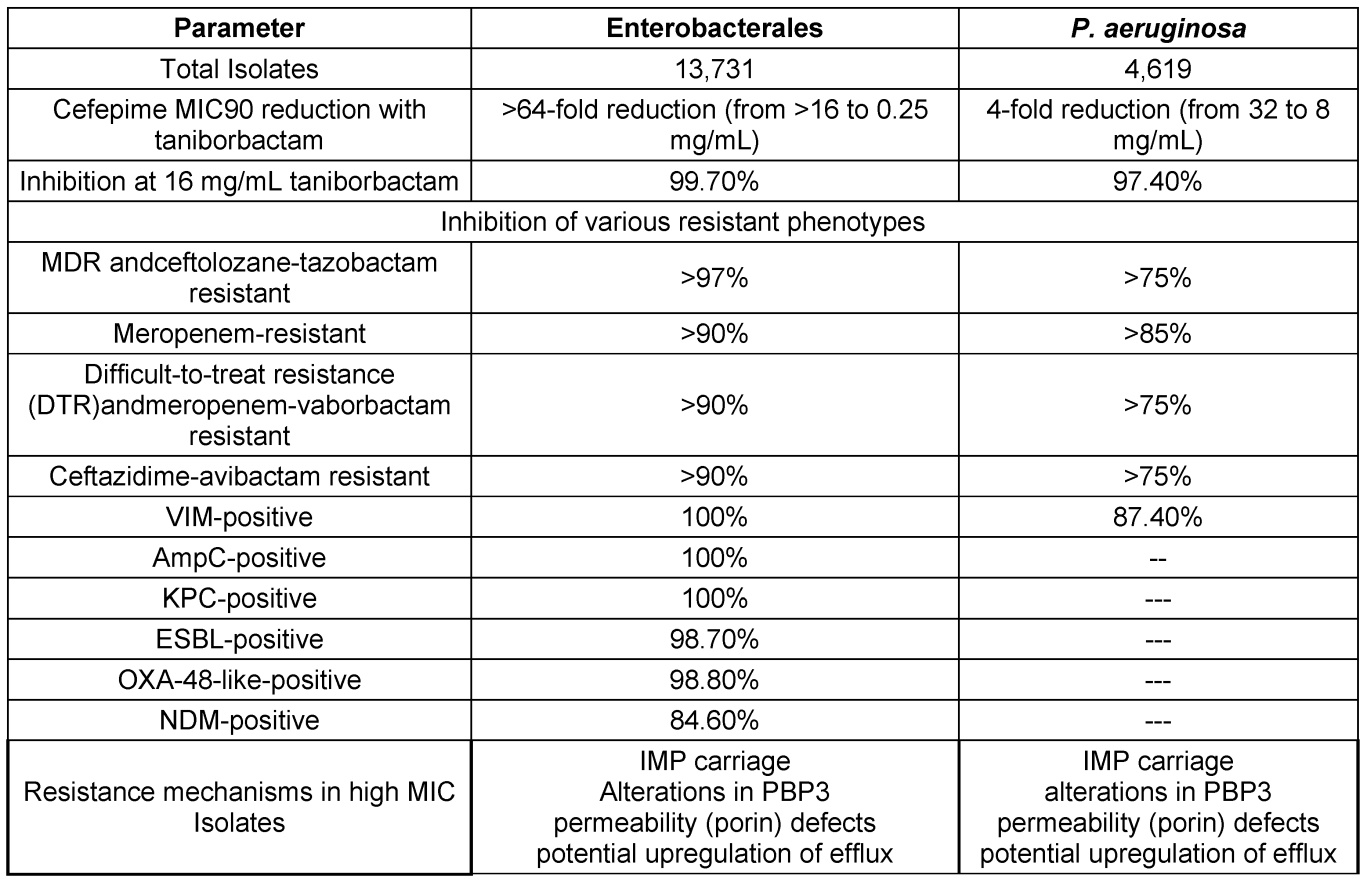
Cefepime-taniborbactam susceptibility in key bacterial populations and resistance profiles (modified according to [97])

**Table 17:**
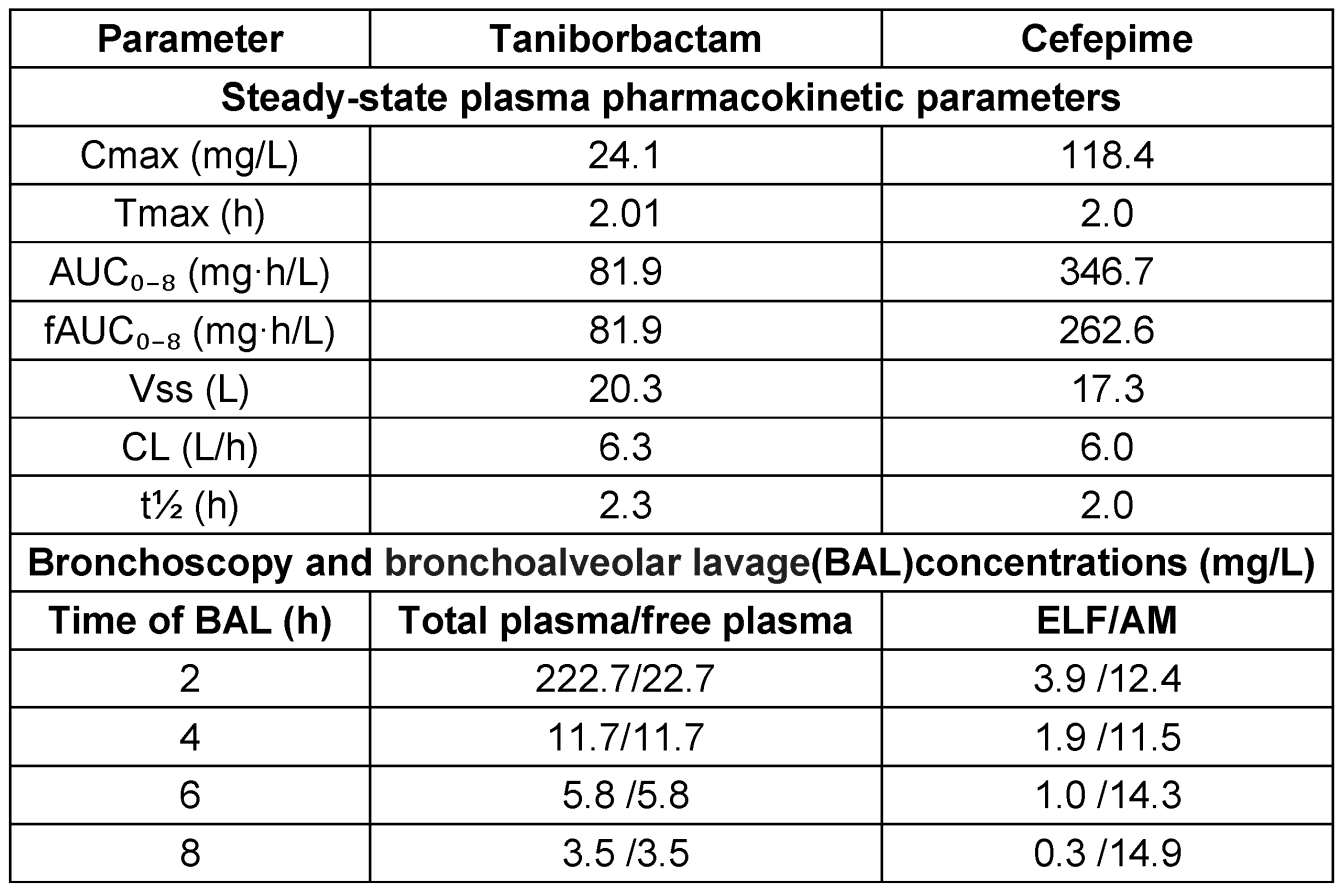
Cefepime-taniborbactam pharmacokinetics in plasma, epithelial lining fluid, and alveolar macrophages from a bronchoscopy study (n=20) (modified according to [101])
